# Research on multimorbidity in primary care. Selected abstracts from the EGPRN meeting in Tampere, Finland, 9–12 May 2019: All abstracts of the conference can be found at the EGPRN website: www.egprn.org/page/conference-abstracts

**DOI:** 10.1080/13814788.2019.1643166

**Published:** 2019-08-16

## INTRODUCTION TO THE THEME OF THE CONFERENCE

Multimorbidity is understood as the coexistence of multiple health conditions in an individual. The number of people suffering from multimorbidity is rising driven by ageing populations and also by the growing burden of non-communicable diseases and mental health problems. Multimorbidity is highly heterogeneous, varying from multiple conditions in frail elderly to combinations of mental health disorders and substance use. Depending on definition, it is estimated that every fourth adult and two in three patients over 65 years of age are multimorbid. In primary care, these estimates are even higher and multimorbidity is a norm among elderly adults.

Multimorbidity is associated with reduced quality of life, impaired functional status, worsened physical and mental health, increased mortality and increased use of health and social care services with associated costs.

