# Supplementary material for: Research on multimorbidity in primary care. Selected abstracts from the EGPRN meeting in Tampere, Finland, 9–12 May 2019: All abstracts of the conference can be found at the EGPRN website: www.egprn.org/page/conference-abstracts
Source: Eur J Gen Pract. 2019 Aug 16;25(3):164–75. doi: 10.1080/13814788.2019.1643166 (PMC6713084; doi:10.1080/13814788.2019.1643166)
Supplement: Related texts

# Abstract: The challenge of multimorbidity for health services and researchers

**DOI:** 10.1080/13814788.2019.1643166

**Published:** 2019-08-16

**Authors:** Bruce Guthrie

**Affiliations:** NHS Research Scotland Primary Care Research Champion, University of Edinburgh, Edinburgh, UK

## Abstract

Multimorbidity challenges existing healthcare organization and research, which remains disease and single-condition focused. Basic science approaches to multimorbidity have the potential to identify important shared mechanisms by which diseases we currently think of as distinct might arise, but there is a pressing need for more applied and health services research to understand better and manage multimorbidity now. There are several recent clinical guidelines, which make recommendations for managing multimorbidity or related issues for patients such as polypharmacy and frailty. However, the evidence base underpinning these recommendations is often weak, and these guidelines, therefore, also help define a research agenda. A key problem for researchers and health services is that multimorbidity is very heterogeneous, in that ‘intermittent low back pain plus mild eczema’ presents very different challenges to researchers and health services compared to ‘active psychosis plus severe heart failure’. Identifying important but tractable research questions is therefore not always straightforward. This presentation will identify important gaps in the evidence, and illustrate how they might be filled. The focus will be on two areas where there is consensus that better evidence is needed to inform care design and delivery: (1) organizational interventions to implement more coordinated and holistic care; and (2) interventions to improve medicines management in people with multimorbidity and polypharmacy. These illustrate both the potential for imaginative research, but also the scale.

# Abstract: Multiprofessional primary healthcare and challenges of multimorbidity

**DOI:** 10.1080/13814788.2019.1643166

**Authors:** Elise Kosunen

**Affiliations:** Faculty of Medicine and Health Technology, Center of Primary Health Care, Pirkanmaa, Finland

## Abstract

Current primary care in Finland is based on the Primary Health Care Act (1972), which addressed numerous new tasks to all municipalities. All of them had to find a new health centre organization, which provides a wide range of health services, including prevention and public health promotion. Multiple tasks require multiprofessional staff, and thus, the Finnish health centre personnel consisted not only of GPs but of public health nurses, midwives, physiotherapists, psychologists, social workers, dentists, etc. During the next decade, there have been some changes but the idea of multiprofessional structure has remained. According to the QUALICOPC study (2012) Finnish GPs are still co-located with several other healthcare professionals compared to most of the European countries; even compared to other Nordic countries which otherwise have many similarities in their primary healthcare. During the last 10 or 15 years, healthcare providers and researchers have recognized a new challenge: our current systems do not meet the needs of patients with multiple health and social problems—and the proportion of these patients is increasing all the time as the population is getting older. One could suppose that preconditions of handling multimorbidity would be excellent in multiprofessional surroundings like ours, but actually, a person with multiple problems is a challenge there, too. Multiprofessional organization in primary care does not guarantee proper care of patients with multiple diseases, if we do not acknowledge the challenge and revise our systems. We have to develop new ways of collaboration and new models of integrated care. The problematic part is secondary care, which is organized with logic of one medical speciality per visit. In Tampere University Hospital district, we have created a care pathway model, which defines the roles of primary healthcare and secondary care. Nationwide, we have recently started to prepare national guidelines for the care of patients with multimorbidity. What we need more in the future is more research on new practices and models.

# Abstract: Violence towards young general practitioners in Croatia remain in silence—a pilot study

**DOI:** 10.1080/13814788.2019.1643166

**Authors:** Iva Jurčević, Marina Oljača Pribanić, Durdica Lazic

**Affiliations:** Health Centre Zagreb-Center, Zagreb, Croatia

## Abstract

**Background:** Workplace violence (WPV) towards healthcare staff is becoming a common problem in different healthcare settings worldwide. Moreover, the prevalence is 16 times higher than in other professions. How often it happened towards young doctors working as general practitioners (GPs) at the beginning of their careers has been rarely studied.

**Research question:** To investigate the frequency and forms of WPV, experienced by the young Croatian GPs from their patients, and violence reporting pattern to the competent institutions.

**Methods:** The cross-sectional study was carried out on 74 GP residents, during their postgraduate study in family medicine in May 2018. A specially designed anonymous questionnaire, developed by Association of Family Physicians of South Eastern Europe, was used to investigate the prevalence and forms of WPV, the narrative description of the traumatic event itself and the process of reporting it.

**Results:** The response rate was 91.9%, female 87%, the median of years working as a GP was 3.5 years. Most of the residents were working in an urban practice (63%), others in the rural and the suburban once (27%, 10%). All GP residents experienced patients’ and caregivers’ violent behaviour directed towards them. High-intensity violence (e.g. physical violence, sexual harassment) was experienced by 44%, middle intensity (e.g. intimidation, visual sexual harassment) by 84% while all residents experienced verbal violence. Only 13.2% residents reported WPV to the competent institutions. Most of GP residents reported the appearance of the new form of violence: the one over the internet.

**Conclusion:** The high prevalence of all types of violence towards young Croatian doctors is worrisome, as is the fact that violent acts are seldom reported to the competent institutions. Those alarming facts could become a threat to GPs career choosing.

# Abstract: Measuring needs and expectations in life when living with multimorbidity—development and validation of the MMQoL scale

**DOI:** 10.1080/13814788.2019.1643166

**Authors:** Kristine Bissenbakker, John Brodersen, Susanne Reventlow, Anne Møller, Alexandra Brandt Ryborg Jønsson

**Affiliations:** Faculty of Health and Medical Sciences, University of Copenhagen, Copenhagen, Denmark

## Abstract

**Background:** The number of people suffering from multiple chronic conditions, multimorbidity, is rising. For society, multimorbidity is known to increase healthcare expenses through more frequent contacts, especially with the primary sector. For the individual, an increasing number of medical conditions are associated with lower quality of life (QoL). However, there is no statistically validated condition-specific patient-reported outcome measure (PROM) for the assessment of QoL among patients with multimorbidity. A validated PROM is essential in order to measure effect in intervention studies for this patient group.

**Research question:** (1) To identify items covering QoL among patients with multimorbidity in a Danish context. (2) To develop and validate a PROM for assessment of QoL among patients with multimorbidity. (3) To utilize the final PROM in a large group of patients with multimorbidity to measure their QoL when living with different combinations and severity of multimorbidity.

**Methods:** Phase 1: qualitative individual and focus group interviews with patients with multimorbidity to identify relevant QoL items. Phase 2: validation of the items through a draft questionnaire sent by email to around 200–400 patients with multimorbidity. Phase 3: psychometric validation of the draft questionnaire securing items with the highest possible measurement quality. Phase 4: assessment of QoL among approximately 2000 patients with multimorbidity from the Danish Lolland–Falster study.

**Results:** There are no results yet. Currently, the interview guide is under development.

**Conclusion:** Despite the rising number of patients with multimorbidity and the known inverse relationship between a patient’s number of medical conditions and their quality of life, there is no statistically validated condition-specific PROM for assessment of QoL among this group. Our aim is that this project’s developed and validated PROM will be used in future intervention studies as a valid measure of QoL among patients with multimorbidity.

# Abstract: Longitudinal multimorbidity patterns in the elderly population using hidden Markov models

**DOI:** 10.1080/13814788.2019.1643166

**Authors:** Marina Guisado-Clavero, Sergio Fernández-Bertolín, Margarita Cabrera-Bean, Albert Roso-Llorach, Quintí Foguet-Boreu, José Valderas, Concepción Violán

**Affiliations:** IDIAP JORDI GOL, Barcelona, Spain

## Abstract

**Background:** Chronic diseases usually have a long duration and slow progression and, as a result, they tend to aggregate in multimorbidity patterns (MPs) during the life course and/or due to shared underlying pathophysiological pathways. Knowledge of how MPs progress over time is necessary to develop effective prevention management strategies.

**Research question:** What are the most likely MPs over time? Which longitudinal shifts from one pattern to another occur during follow-up?

**Methods:** A prospective longitudinal study based on electronic health records was conducted during 2012–2016 in Catalonia, Spain. For people aged ≥65 years, we extracted data on demographics and diagnostic codes for chronic diseases (ICD-10). Machine-learning techniques were applied for the identification of disease clusters using fuzzy c-means analysis to obtain initial clusters. To estimate longitudinal MPs and their progression for each individual a hidden Markov model was fitted, estimating: (1) the transition probability matrix between clusters; (2) the initial cluster probability; (3) the most likely trajectory for each individual. The prevalence of disease in each cluster, observed/expected ratios (O/E ratios) and disease exclusivity was determined for each MP. Criteria used to designate cluster: O/E ratio ≥2.

**Results:** In total, 916 619 individuals were included. Ten MPs were identified. The cluster including the most prevalent diseases was designated non-specific (42.0% of individuals). The remaining nine clusters included the following anatomical systems: ophthalmologic and mental diseases (19.3%), osteometabolic (7.9%), cardio-circulatory (6.6%), and others. Most patients, minimum 59.2%, remained in the same cluster during the study period. The highest transitions to the mortality state were observed in the cardio-circulatory (37.1%) and nervous (31.8%) MPs.

**Conclusion:** Ten significant longitudinal MPs were found. The application of sophisticated statistical techniques ideally suited the study of the MPs and allowed for characterization over time. This method is useful to establish a probabilistic evolution of MPs.

# Abstract: Barriers and enablers to deprescribing in older patients with multimorbidity and polypharmacy

**DOI:** 10.1080/13814788.2019.1643166

**Authors:** Zsofia Rozsnyai, Katharina Tabea Jungo, Emily Reeve, Arnaud Chiolero, Rosalinde Ke Poortvliet, Nicolas Rodondi, Jacobijn Gussekloo, Sven Streit

**Affiliations:** Institute of Primary Health Care (BIHAM), Bern, Switzerland

## Abstract

**Background:** Multimorbidity and polypharmacy have become the norm for general practitioners (GPs). Ideally, GPs search for inappropriate medication and, if necessary, deprescribe. However, it remains challenging to deprescribe given time constraints and little backup from guidelines. Furthermore, barriers and enablers to deprescribing among patients have to be accounted for.

**Research question:** To identify barriers and enablers to deprescribing in older patients with polypharmacy.

**Methods:** We surveyed among patients >70 years with multimorbidity (>2 chronic conditions) and polypharmacy (>4 regular medicines). We invited Swiss GPs to recruit eligible patients, each of whom completed a paper-based survey on demography, medications and chronic conditions. We applied the revised patients’ attitudes towards deprescribing (rPATD) questionnaire and added 12 additional questions and two open questions to assess barriers and enablers towards deprescribing.

**Results:** We analysed the first 221 responses received so far and full results will be presented at the conference. Participants were 79.3 years in mean (SD 5.8) and 48% female. Thirty-one percent lived alone, and 85% prepared their medication themselves, all others required help. Seventy-six percent of participants took 5–9 regular medicines and 24% took ≥10 up to 22 medicines. Participants (76%) were willing to deprescribe one or more of their medicines and 78% did not have any negative experience with deprescribing. Age and gender were not associated with their willingness to deprescribe. Important barriers to deprescribing were satisfaction with drugs (96%), long-term drugs (56%) and noticing positive effects when taking them (92%). When it comes to deprescribing, 89% of participants wanted as much information as possible on their medicines. Having a good relationship with their GP was a further key factor to them (85%).

**Conclusion:** Most older adults are willing to deprescribe. They would like to be informed about their medicines and want to discuss deprescribing to achieve shared decision-making with the GP they trust.

# Abstract: Comorbidity in family medicine—causal or casual? What is the effect of illness diversity? A longitudinal observational study in primary care

**DOI:** 10.1080/13814788.2019.1643166

**Authors:** Jean Karl Soler, Inge Okkes, Nicola Buono

**Affiliations:** MIPC, Rabat, Malta

## Abstract

**Background:** A study of casual versus causal comorbidity in family medicine in three practice populations from the Netherlands, Malta and Serbia.

**Research question:** (1) What is the observed comorbidity of the 20 most common episodes of care in three countries? (2) How much of the observed comorbidity is likely to be casual versus causal?

**Methods:** Participating family doctors (FDs) in the Netherlands, Malta and Serbia recorded details of all patient contacts in an episode of care structure using electronic medical records based on the International Classification of Primary Care, collecting data on all elements of the doctor–patient encounter, including the diagnostic labels (episode of care labels, EoCs). Comorbidity was measured using the odds ratio of both conditions being incident or rest-prevalent in the same patient in one-year data frames, as against not.

**Results:** Comorbidity in family practice expressed as odds ratios between the 41 most prevalent (joint top 20) episode titles in the three populations. Specific associations were explored in different age groups to observe the changes in odds ratios with increasing age as a surrogate for a temporal or biological gradient.

**Conclusion:** After applying accepted criteria for testing the causality of associations, it is reasonable to conclude that most of the observed primary care comorbidity is casual. It would be incorrect to assume causal relationships between co-occurring diseases in family medicine, even if such a relationship might be plausible or consistent with current conceptualizations of the causation of disease. Most observed comorbidity in primary care is the result of increasing illness diversity.

# Abstract: Quality of life among older adults living with multimorbidity: Findings from the European SHARE database

**DOI:** 10.1080/13814788.2019.1643166

**Authors:** Tatjana T. Makovski, Beatriz Poblador Plou, Maria Ruiz-Castell, Saverio Stranges, Laetitia Huiart, Maurice P. Zeegers, Marjan Van Den Akker

**Affiliations:** Luxembourg Institute of Health, Luxembourg

## Abstract

**Background:** Multimorbidity prevalence increases with age while declining quality of life (QoL) is one of its major consequences.

**Research question:** The study aims to: (1) Assess the relationship between increasing number of diseases and QoL. (2) Identify the most frequently occurring patterns of diseases and how they relate to QoL. (3) Observe how these associations differ across different European countries and regions.

**Methods:** Cross-sectional data analysis performed on wave six of the population-based survey of health, ageing and retirement in Europe (SHARE) (*n* = 68 231). Data were collected in 2015 among population 50+ years old in 17 European countries and Israel. Multimorbidity is defined as the co-occurrence of two or more chronic conditions. Conditions were self-declared and identified through an open-end questionnaire containing 17 prelisted conditions plus conditions added by participants. Control, autonomy, self-realization and pleasure questionnaire (CASP-12v) was used to evaluate QoL. Association between increasing number of diseases and QoL was assessed with linear regression. Factor analysis is being conducted to identify patterns of diseases to evaluate their impact on QoL further. Multilevel analysis will take into account differences between countries and regions. Confounding was searched with directed acyclic graph (DAG) method and included age, sex, education, socio-economic status, behavioural habits, social support and healthcare parameters.

**Results:** Participants (49.09%) had two or more diseases. Maximum number of diseases per person was 13, mean number was 1.9. Unadjusted preliminary analysis showed that on average QoL decreases by −1.27 (95%CI: −1.29, −1.24) with each added new condition across Europe. The decline appears to be the steepest in Spain, −1.61 (95%CI: −1.71, −1.51), and the least so in Israel, −0.67 (95%CI: −0.82, −0.52).

**Conclusion:** Ongoing analysis will identify disease patterns, which may have the highest impact on QoL, as well as to elucidate the role of confounders in the relationship between increasing number of diseases and disease patterns with QoL.

# Abstract: High number of GP visits and number of illnesses are risk factors for multimorbid patients

**DOI:** 10.1080/13814788.2019.1643166

**Authors:** Jerome Fonseca, Jeanlin Viala, Anne Lise Thos, Delphine Le Goff, Sophie Lalande, Michele Odorico, Pierre Barraine, Patrice Nabbe, Jean Yves Le Reste

**Affiliations:** EA SPURBO, Médecine Générale, Brest, France

## Abstract

**Background:** Through a systematic review of the literature and qualitative research across Europe, the European General Practitioners Research Network (EGPRN) has designed and validated a comprehensive definition of multimorbidity. It is a concept considering all the biopsychosocial conditions of a patient. This concept encompasses more than 50 variables and is consequently difficult to use in primary care. Consideration of adverse outcomes (such as death or acute hospitalization) could help to distinguish which variables could be risk factors of decompensation within the definition of multimorbidity.

**Research question:** Which criteria in the EGPRN concept of multimorbidity could detect outpatients at risk of death or acute hospitalization (i.e. decompensation) in a primary care cohort at 24-months of follow-up?

**Methods:** Primary care outpatients (131) answering to EGPRN’s multimorbidity definition were included by GPs, during two periods of inclusion in 2014 and 2015. At 24 months follow-up, the status ‘decompensation’ or ‘nothing to report’ was collected. A logistic regression following a Cox model was performed to achieve the survival analysis and to identify potential risk factors.

**Results:** At 24 months follow-up, 120 patients were analysed. Three different clusters were identified. Forty-four patients, representing 36.6% of the population, had either died or been hospitalized more than seven consecutive days. Two variables were significantly associated with decompensation: Number of GPs encounters per year (HR: 1.06; 95%CI: 1.03–1.10, *p* <0.001), and total number of diseases (HR: 1.12; 95%CI: 1.03–1.33; *P* = 0.039).

**Conclusion:** To prevent death or acute hospitalization in multimorbid outpatients, GPs may be alert to those with high rates of GP encounters or a high number of illnesses. These results are consistent with others in medical literature.

# Abstract: Bringing together older multimorbid patients with polypharmacy, general practitioners, and eHealth: Protocol of a cluster randomized controlled trial in Swiss primary care

**DOI:** 10.1080/13814788.2019.1643166

**Authors:** Katharina Tabea Jungo, Zsofia Rozsnyai, Carmen Floriani, Sven Streit

**Affiliations:** Institute of Primary Health Care (BIHAM), University of Bern, Bern, Switzerland

## Abstract

**Background:** Polypharmacy and multimorbidity are on the rise. Consequently, general practitioners (GPs) treat an increasing number of multimorbid patients with polypharmacy. To limit negative health outcomes, GPs should search for inappropriate medication intake in such patients. However, systematic medication reviews are time-consuming. Recent eHealth tools, such as the ‘systematic tool to reduce inappropriate prescribing’ (STRIP) assistant, provide an opportunity for GPs to get support when conducting such medication reviews.

**Research question:** Can the STRIP assistant as electronic decision support help GPs to optimize medication appropriateness in older, multimorbid patients with polypharmacy?

**Methods:** This cluster randomized controlled trial is conducted in 40 Swiss GP practices, each recruiting 8–10 patients aged ≥65 years, with ≥3 chronic conditions and ≥5 chronic medications (320 patients in total). We compare the effectiveness of using the STRIP assistant for optimizing medication appropriateness to usual care. The STRIP assistant is based on the STOPP/START criteria (version 2) and, for this trial, it is implemented in the Swiss eHealth setting where some GPs already share routine medical data from their electronic medical records in a research database (FIRE). Patients are followed-up for 12 months and the change in medication appropriateness is the primary outcome. Secondary outcomes are the numbers of falls and fractures, quality of life, health economic parameters, patients’ willingness to deprescribe as well as implementation barriers and enablers for GPs when using the STRIP assistant.

**Results:** Patient recruitment started in December 2018. This presentation focuses on the study protocol and the challenges faced when testing this new software in Swiss primary care.

**Conclusion:** Finding out whether the STRIP assistant is an effective tool and beneficial for older and multimorbid patients, who are usually excluded from trials, will have an impact on the coordination of chronic care for multimorbid patients in Swiss primary care in this new eHealth environment.

# Abstract: Lifetime risk, multimorbidity and disease-free life expectancy of non-communicable diseases in the general population: A prospective cohort study

**DOI:** 10.1080/13814788.2019.1643166

**Authors:** Silvan Licher, Alis Heshmatollah, Kimberly D. Van Der Willik, Bruno H. Ch. Stricker, Rikje Ruiter, Emmely W. De Roos, Lies Lahousse, Peter J. Koudstaal, Albert Hofman, Lana Fani, Guy G. O. Brusselle, Daniel Bos, Banafsheh Arshi, Maryam Kavousi, Maarten J. G. Leening, M. Kamran Ikram, M. Arfan Ikram

**Affiliations:** Erasmus MC—University Medical Center Rotterdam, Rotterdam, The Netherlands

## Abstract

**Background:** The burden and preventive potential of disease is typically estimated for each non-communicable disease (NCD) separately but NCDs often co-occur, which hampers reliable quantification of their overall burden and joint preventive potential in the population.

**Research questions:** What is the lifetime risk of developing any NCD? Which multimorbidity clusters of NCDs cause the greatest burden? To what extent do three key shared risk factors, namely smoking, hypertension and being overweight, influence this risk, life-expectancy and NCD-multimorbidity?

**Methods:** Between 1990 and 2012 we followed NCD-free participants aged ≥45 years at baseline from the Dutch prospective Rotterdam study for incidents of stroke, heart disease, diabetes, chronic respiratory disease, cancer, and neurodegenerative disease. We quantified (co-)occurrence and remaining lifetime risk of NCDs in a competing risk framework, and studied the effects of smoking, hypertension, and being overweight on lifetime risk and life expectancy.

**Results:** During follow-up of 9061 participants, 814 participants were diagnosed with stroke, 1571 with heart disease, 625 with diabetes, 1004 with chronic respiratory disease, 1538 with cancer, and 1065 with neurodegenerative disease. Among those, 1563 participants (33.7%) were diagnosed with multiple diseases. The lifetime risk of any NCD from the age of 45 onwards was 94.0% (95%CI: 92.9–95.1) for men and 92.8% (95%CI: 91.8–93.8) for women. Absence of shared risk factors was associated with a 9.0-year delay (95%CI: 6.3–11.6) in the age at onset of any NCD. Furthermore, overall life expectancy for participants without risk factors was 6.0 years (95%CI: 5.7–7.9) longer than those with these risk factors. Participants without these risk factors spent 21.6% of their remaining lifetime with NCDs, compared to 31.8% for those with risk factors.

**Conclusion:** Nine out of 10 individuals aged 45 years and older will develop at least one NCD during their remaining lifetime. A third was diagnosed with multiple NCDs during follow-up. Absence of three common shared risk factors related to compression of morbidity of NCDs.

# Abstract: Multimorbidity and polypharmacy in Canada: Examining prevalence and patterns in primary healthcare using a national electronic medical record database

**DOI:** 10.1080/13814788.2019.1643166

**Authors:** Kathryn Nicholson, Martin Fortin, Lauren Griffith, Amanda Terry, Tyler Williamson, Dee Mangin, Saverio Stranges

**Affiliations:** Western University, Woodstock, Canada

## Abstract

**Background:** The accumulation of multiple chronic diseases (multimorbidity) and multiple prescribed medications (polypharmacy) over time may influence the extent to which an individual maintains health and well-being in later life.

**Research question:** This research aims to describe the patterns (sequence and timing) of multimorbidity and polypharmacy that accumulate over time among primary healthcare patients in Canada.

**Methods:** Data are derived from the Canadian primary care sentinel surveillance network (CPCSSN) electronic medical record (EMR) database that holds ≥1 million longitudinal, de-identified records. Multimorbidity will be identified with 20 categories, cut-off points of ≥2 and ≥3 chronic conditions and the International Classification of Disease (ICD) classification system. Polypharmacy will be identified using the cut-off points of ≥5 and ≥10 medication classes and the Anatomical Therapeutic Chemical (ATC) classification system. Analyses will be conducted using Java and Stata 14.2 software.

**Results:** The prevalence of chronic diseases and prescribed medications will be presented, as well as the patterns that are observed among adults and older adults in Canada. The most frequent patterns (combinations and permutations) of multimorbidity and polypharmacy will be presented, stratified by sex and age category. The relationships with other factors, such as the presence of frailty, disability or increased health service use, will be examined. As well, the methodological challenges to identifying the presence and sequence of multimorbidity and polypharmacy in national, longitudinal data will be discussed.

**Conclusion:** This research will explore the profiles of multimorbidity and polypharmacy in mid- and late-life using a national, longitudinal database. These findings can be used strategically to inform healthcare delivery and to contribute to the understanding of multimorbidity and polypharmacy in the international literature. Reducing the burden of prescribed medications and the harms of polypharmacy are key tasks within the context of multimorbidity.

# Abstract: Implementation of video recording with immediate feedback in real-time consultation in general practice

**DOI:** 10.1080/13814788.2019.1643166

**Authors:** Tan-Trung Phan, Younes Touati, Sophie Brossier, Monica Neagoe, Florence Adeline-Duflot, Badr Eddine Hicheur, Laurence Compagnon, Emilie Ferrat

**Affiliations:** Department of Education and Research in General Practice, Paris-Est Créteil University, Créteil, France

## Abstract

**Background:** Immediate feedback is underused in the French medical education curriculum, specifically with video-recorded consultation.

**Research question:** The objective of this study was to evaluate the feasibility and the interest in this teaching method as a training and assessment tool in the learning process of general practitioner (GP) trainees.

**Methods:** During the period November 2017 to October 2018, trainees in ambulatory training courses collected quantitative data about recording consultations with a video camera: numbers of recordings, feedback, patients’ participation refusals, and information about the learning process and competencies. The trainees’ level of satisfaction was measured by means of a questionnaire at the end of their traineeship.

**Results:** Sixty-seven trainees were recruited and 44 of them 65.7% actively participated in the study; 607 video recordings and 243 feedback with trainers were performed. Few patients (18.5%) refused the video-recording. Most trainees considered video recording with immediate feedback to be a relevant learning tool. It made it possible for the participants to observe their difficulties and their achievements. ‘Relation, communication, patient-centred care’ was the most built competency, non-verbal communication, in particular. Time was the main limiting factor of this teaching method. Most trainees were in favour of its generalization in their university course.

**Conclusion:** Video recording with immediate feedback in real-time consultation needs to be adapted to training areas and depends on time and logistics. This teaching method seems to be useful in the development of communication skills. It could lift the barriers of the trainer’s physical presence near GP trainees during immediate feedback in real-time consultation. It could help trainees to build their competencies while enhancing the place of immediate feedback in the general practice curriculum. It could also constitute an additional tool for the certification of GP trainees.

# Abstract: How does child abuse suspicion arise in general practice?

**DOI:** 10.1080/13814788.2019.1643166

**Authors:** Erik Stolper, Margje Van De Wiel

**Affiliations:** Maastricht University, General Practice, Heerde, The Netherlands

## Abstract

**Background:** Child abuse is widespread, occurs in all cultures and communities and remains undiscovered in 90% of the cases. In total, 80% of reported child abuse concerns emotional ill-treatment. In the Netherlands, at least 3% (118 000) of children are victims of child abuse resulting in 50 deaths each year. Only 1–3% of abuse cases are reported by general practitioners (GPs) to the Child Protective Services agency (CPS). To explain this low reporting rate, we examined GPs’ experiences with child abuse.

**Research question:** How does the suspicion of child abuse arise in GPs’ diagnostic reasoning? How do they act upon their suspicion and what kind of barriers do they experience in their management?

**Methods:** In total 26 GPs (16 female) participated in four focus groups. We used purposive sampling to include GPs with different levels of experience in rural and urban areas spread over the Netherlands. We used NVivo for thematic content analysis.

**Results:** Suspected child abuse arose based on common triggers and a gut feeling that ‘something is wrong here’. GPs acted upon their suspicion by gathering more data by history taking and physical examination. They often found it challenging to decide whether a child was abused because parents, despite their good intentions, may lack parenting skills and differ in their norms and values. GPs reported clear signs of sexual abuse and physical violence to CPS. However, in less clear-cut cases they followed-up and built a supporting network around the family. Most GPs highly valued the patient–doctor relationship while recognizing the risk of pushing boundaries.

**Conclusion:** A low child abuse reporting rate by GPs to CPS does not mean a low detection rate. GPs use patients’ trust in their doctor to improve a child’s situation by involving other professionals.

# Abstract: Eleven quality of life scales are available for the general population, nevertheless very few are reproducible and none have undertaken an external validation survey

**DOI:** 10.1080/13814788.2019.1643166

**Authors:** Jeanlin Viala, Jerome Fonseca, Le Goff Delphine, Lalande Sophie, Michele Odorico, Laurent Tosoni, Paul Aujoulat, Lucas Beurton-Couraud, Pierre Barraine, Patrice Nabbe, Jean Yves Le Reste

**Affiliations:** EA SPURBO, Médecine Générale, Brest, France

## Abstract

**Background:** Quality of life is an essential theme for quantitative surveys in primary care. Treatments and procedures need to be assessed on whether they change patients’ quality of life. This has led to the creation of evaluation scales. The purpose of this study was to determine reproducibility and efficiency of 11 previously selected quality of life scales (selected with a systematic review) for the general population.

**Research question:** What is the best possible reproducible and efficient quality of life scale for the general population?

**Methods:** The search was conducted from November 2017 to April 2018 in PubMed and Cochrane databases, according to the PRISMA (preferred reporting items for systematic reviews and meta-analyses) protocol. The inclusion criteria were the psychometric qualities for each of the 11 scales studied. Articles dealing with subpopulations or those not written in IMRAD format were excluded. The collected values were reproducibility and efficiency.

**Results:** Out of 206, 46 selected articles were included. Cronbach’s alpha by domain and Pearson’s coefficient were the most analysed psychometrics. No valid efficiency data was obtained. The internal consistency was over 0.7 for the SF-36, SF12v2 and EQ-5D scales. The Pearson coefficient was over 0.4 for the SF36v2, SF-12 and SF-12v2 scales. The Cohen’s kappa ranged from 0.4 to 0.80 for the EQ-5D questionnaire.

**Conclusion:** No scale is fully validated. Reproducibility values were incomplete (Cronbach’s alpha and Pearson’s most expressed). No efficiency data was found. The most validated scales are the SF family and the EQ-5D. Researchers and clinicians should be aware of these limitations when choosing a quality of life scale. They should return to the scales’ designs to choose the one that underlines the type of quality of life they want to assess as no external validity is available.

# Abstract: Comparison of patient enablement instrument to two single-item measures

**DOI:** 10.1080/13814788.2019.1643166

**Authors:** Elina Tolvanen, Tuomas Koskela, Elise Kosunen

**Affiliations:** General Practice, Tampere, Pirkanmaa, Finland

## Abstract

**Background:** The patient enablement instrument (PEI) is an established patient-reported outcome measure (PROM) that reflects the quality of a GP appointment. It is a six-item questionnaire, addressed to the patient immediately after a consultation.

**Research question:** The study aimed to evaluate whether a single-item measure (the Q1), based on the PEI, or a single question extracted from the PEI itself (the Q2) could replace the PEI when measuring patient enablement among Finnish healthcare centre patients.

**Methods:** The study design included (1) a pilot study with brief interviews with the respondents, (2) a questionnaire study before and after a single appointment with a GP, and (3) a telephone interview two weeks after the appointment. The correlations between the measures were examined. The sensitivity, specificity and both positive and negative predictive values for the Q1 and the Q2 were calculated, with different PEI score cut-off points.

**Results:** Altogether 483 patients with completed PEIs were included in the analyses. The correlations between the PEI and the Q1 or the Q2 were 0.48 and 0.84, respectively. Both the Q1 and the Q2 had high sensitivity and negative predictive value in relation to patients with lower enablement scores. The reliability coefficients were 0.24 for the Q1 and 0.76 for the Q2.

**Conclusion:** The Q2 seems to be a valid and reliable way to measure patient enablement. The Q1 seems to be less correlated with the PEI, but it also has high negative predictive value in relation to low enablement scores.

# Abstract: The effect of electronic reminders on the recording of diagnoses in primary care: A longitudinal follow-up study

**DOI:** 10.1080/13814788.2019.1643166

**Authors:** Tuomo Lehtovuori

**Affiliations:** Tampere University, Helsinki, Finland

## Abstract

**Background:** This study examined if using electronic reminders increases the rate of diagnosis recordings in the patient chart system following visits to a general practitioner (GP). The impact of electronic reminders was studied in the primary care of a Finnish city.

**Research question:** How effective is the reminder of the information system in improving the diagnostic level of primary care? Which is better and how: financial incentives or reminders?

**Methods:** This was an observational retrospective study based on a before-and-after design and was carried out by installing an electronic reminder in the computerized patient chart system to improve the recording of diagnoses during GP visits. The quality of the recorded diagnoses was observed before and after the intervention. The effect of this intervention on the recording of diagnoses was also studied.

**Results:** Before intervention, the level of recording diagnoses was about 40% in the primary care units. After four years, the recording rate had risen to 90% (*p* < 0.001). The rate of change in the recording of diagnoses was highest during the first year of intervention. In the present study, most of the visits concerned mild respiratory infections, elevated blood pressure, low back pain and type II diabetes.

**Conclusion:** An electronic reminder improved the recording of diagnoses during the visits to GPs. The present intervention produced data, which reflects the distribution of diagnoses in real clinical life in primary care and thus provides valid data about the public.

# Abstract: Feasibility of a checklist in treating hypertension in primary care—baseline results from a cluster-randomized controlled trial (check and support)

**DOI:** 10.1080/13814788.2019.1643166

**Authors:** Aapo Tahkola, Päivi Korhonen, Hannu Kautiainen, Teemu Niiranen, Pekka Mäntyselkä

**Affiliations:** Health Centre of Jyväskylä Cooperation Area, Jyväskylä, Finland; Institute of Public Health and Clinical Nutrition, University of Eastern Finland, Kuopio, Finland

## Abstract

**Background:** Most patients with antihypertensive medication do not achieve their blood pressure (BP) target. Several barriers to successful hypertension treatment are well identified but we need novel ways of addressing them.

**Research question:** Can using a checklist improve the quality of care in the initiation of new antihypertensive medication?

**Methods:** This non-blinded, cluster-randomized, controlled study was conducted in eight primary care study centres in central Finland, randomized to function as either intervention (*n* = 4) or control sites (*n* = 4). We included patients aged 30–75 years who were prescribed antihypertensive medication for the first time. Initiation of medication in the intervention group was carried out with a nine-item checklist, filled in together by the treating physician and the patient. The treating physician managed hypertension treatment in the control group without a study-specific protocol.

**Results:** In total, 119 patients were included in the study, of which 118 were included in the analysis (*n* = 59 in the control group, *n* = 59 in the intervention group). When initiating medication, an adequate BP target was set for 19% of the patients in the control group and for 68% in the intervention group. Shortly after the appointment, only 14% of the patients in the control group were able to remember the adequate BP target, compared with 32% in the intervention group. The use of the checklist was also related to more regular agreement on the next follow-up appointment (64% in the control group vs 95% in the intervention group).

**Conclusion:** Even highly motivated new hypertensive patients in Finnish primary care have significant gaps in their treatment-related skills. The use of a checklist for initiation of antihypertensive medication was related to substantial improvement in these skills. Based on our findings, the use of a checklist might be a practical tool for clinicians initiating new antihypertensive medications.

# Abstract: Glycaemic control, use of steroids and infection among patients with type 2 diabetes

**DOI:** 10.1080/13814788.2019.1643166

**Authors:** Anthony Heymann, Galia Zacai

**Affiliations:** University Tel Aviv, Ramat Gan, Israel

## Abstract

**Background:** Previous studies have shown an increased rate of infection among patients with diabetes; however, it is unclear from these studies if the level of HbA1c is correlated with infection.

**Research question:** This study aimed to examine the association between glycaemic control of type 2 diabetes patients and the incidence of infections.

**Methods:** An HMO database was used to identify all DM patients. The first HbA1c test during the period of the study was selected for each patient; then an infection diagnosis was searched in the 60 days that followed the test. We compared the HbA1c test results that were followed by an infection to those that were not. After applying exclusion criteria: having cancer, receiving immunosuppressive medication, undergoing dialysis treatment, anaemia less than 9 mg%, and G6PD deficiency, there remained 33 637 patients in the cohort. The study period was October 2014 to September 2017. The following information was collected: age, gender, socio-economic index, BMI, use of hypoglycaemic and steroid medication in the 90 days before infection, and comorbid conditions (IHD, PVD, CVA, CCF, asthma, COPD, Parkinson’s disease, dementia, CRF).

**Results:** In total, 804 patients had an infection within 60 days following an HbA1c test. For cellulitis, cholecystitis, herpes zoster, pneumonia and sinusitis the HbA1c was higher than those patients that had no infection (for cellulitis 7.603 vs 7.243). When factored into logistic regression analysis, we found that other chronic diseases increased the risk of infection between 29 and 60%. Each increase of a gram of HbA1c increased the risk by 8.5%. Use of steroids in the 90 days before the infection increases the chance of infection by 734%.

**Conclusion:** Increasing HbA1c and comorbidity both increase the risk of infection among type 2 diabetics but use of oral or injectable steroids is a much more significant risk factor.

# Abstract: The TATA survey: The translations of the WAI SR are homogeneous between Spain, Poland, Slovenia, France and Italy

**DOI:** 10.1080/13814788.2019.1643166

**Authors:** Fabienne Morvan, Nicolas Toupin, Krzysztof Buczkowski, Ana Claveria, Nicola Buono, Davorina Petek, Jeanlin Viala, Jerome Fonseca, Jean Yves Le Reste

**Affiliations:** DUMG Brest, Brest, France

## Abstract

**Background:** The concept of therapeutic alliance emerged in the beginning of the twentieth century and came from psychoanalysis. This notion was then extended to the somatic field and aims to replace the paternalistic model in the doctor–patient relationship. The EGPRN TATA group selected the WAI SR as the most reliable and reproducible scale to assess therapeutic alliance. To use it within Europe, it was necessary to translate it into most European languages. The following study aimed to assess the linguistic homogeneity of five of these translations.

**Research question:** Are the translations of the WAI SR homogeneous between Spain, Poland, Slovenia, France and Italy?

**Methods:** Forward-backward translations were achieved in five participating countries (Spain, Poland, France, Slovenia and Italy). Using a Delphi procedure, a global homogeneity check was then performed by comparing the five backward translations during a physical meeting involving GP teachers/researchers from many European countries; the heterogeneity of the participants’ origins was a token of reliability.

**Results:** In the assessment of the five translations, 107 experts participated. A consensus was obtained in one to two Delphi rounds for each. During the ‘homogeneity check,’ some discrepancies were noted with the original version and were discussed with the local teams. This last stage permitted to highlight cultural discrepancies and real translation issues and to correct if needed.

**Conclusion:** Five homogeneous versions of the WAI SR are now available in five European languages. They will be helpful to evaluate therapeutic alliance at different levels: for GPs in daily practice, for students during the initial and continuous training, and for further research in these five countries.

# Abstract: Associations of sociodemographic and clinical factors with perinatal depression among Israeli women

**DOI:** 10.1080/13814788.2019.1643166

**Authors:** Limor Adler, Judith Tsamir, Rachel Katz, Gidon Koren, Ilan Yehoshua

**Affiliations:** Israel Association of Family Physicians, Ashkelon, Israel

## Abstract

**Background:** Perinatal depression has been associated with psychiatric morbidity in mothers and their offspring. This study assessed the prevalence of perinatal depressive symptoms in a large population of women and investigated associations of these symptoms with demographic and clinical factors.

**Research question:** Which factors (including sociodemographic, medical, lifestyle, and laboratory test) are associated with perinatal depression?

**Methods:** All members of Maccabi Health Services who completed the Edinburgh Postnatal Depression Scale (EPDS) during 2015–2016 were included in the study. Odds ratios (ORs) were calculated for associations of sociodemographic, medical, lifestyle, and laboratory test factors with perinatal depressive symptoms, according to a score >10 on the EPDS.

**Results:** Of 27 912 women who filled the EPDS, 2029 (7.3%) were classified as having peripartum depression. In a logistic regression analysis, the use of antidepressant medications, particularly for a period greater than three months, Arab background, current or past smoking, a diagnosis of chronic diabetes and age under 25 years were all associated with increased ORs for perinatal depression; while Orthodox Jewish affiliation, residence in the periphery and higher haemoglobin level were associated with lower ORs. Incidences of depression were 17.4% in women with a history of antidepressant medication, 16% among women with diabetes, and 11.8% among current smokers.

**Conclusion:** Several demographic, medical, and lifetime factors were found to be substantially more prevalent among women with symptoms of perinatal depression than those without. Encouraging women to complete the EPDS during and following pregnancy may help identify women in need of support.

# Abstract: Identifying high-need patients with multimorbidity based on their primary care medical records

**DOI:** 10.1080/13814788.2019.1643166

**Authors:** Marianne Heins, Mieke Rijken, José Valderas, François Schellevis, Joke Korevaar

**Affiliations:** Nivel, Utrecht, The Netherlands

## Abstract

**Background:** With growing populations of patients with multimorbidity, general practitioners need insight into which patients in their practice are most in need for person-centred integrated care (‘high-need’ patients). Using data from electronic primary care medical records to automatically create a list of possible ‘high need’ patients could be a quick and easy first step to assist GPs in identifying these patients.

**Research question:** Can ‘high need’ patients with multimorbidity be identified automatically from their primary care medical records?

**Methods:** Pseudonymized medical records of patients with multimorbidity (≥2 chronic diseases) were analysed. Data was derived from the Nivel primary care database, a large registry containing data routinely recorded in electronic health records. This includes data on healthcare use, health problems and treatment. Logistic regression analysis was conducted to predict outcomes (frequent contact with the general practice, ER visits and unplanned hospital admissions). Predictors were age, sex, healthcare use in the previous year, morbidity and medication use.

**Results:** In total, 245 065 patients with multimorbidity were identified, of which 48% were above the age of 65 and 57% female. More than 42% had five GP contacts in the previous year and 62% used five or more different medications. Frequent contact with the general practice could be reliably predicted using only the number of contacts in the previous year (AUC: 0.82). Adding all other predictors (including specific chronic conditions) only improved the predictive value of the model marginally (AUC: 0.84). Identifying patients with a high risk for ER visits and unplanned hospital admissions proved more difficult (AUC: 0.67 and 0.70, respectively).

**Conclusion:** ‘High need’ patients with multimorbidity can be automatically selected from primary care medical records using only the number of contacts with the general practice in the previous year. Composing a list of these patients can help GPs to identify those eligible for person-centred integrated care.

# Abstract: Self-reported adherence in primary care: A systematic review

**DOI:** 10.1080/13814788.2019.1643166

**Authors:** Delphine Rubé, Cindy Rabot

**Affiliations:** DUMG Tours, Faculté de Médecine, Sancoins, France

## Abstract

**Background:** About 50% of patients adhere to chronic therapy in France. Improving adherence should improve their care. Identifying the patient’s difficulties in taking medication is complex for the physician, because there is no gold standard for measuring adherence to medications. How can the general practitioner in his/her practice identify patient compliance?

**Research question:** Analyse studies that develop or validate scales used to estimate adherence in primary care.

**Methods:** A systematic review of the literature from PubMed, the Cochrane Library and PsycINFO databases. The search terms used were the MeSH terms (or adapted to the database’s vocabulary): questionnaire, compliance and primary care. All articles were retained whatever the language of writing. Selection criteria were: assessment of the development, validation or reliability of one or more compliance scales; taking place in primary care. One reviewer screened titles, which included the term adherence then abstracts and full text. Only articles evaluating the development, validity or reliability of a primary care adherence rating scale were included in analysis.

**Results:** In total 1022 articles were selected and 18 articles were included. Seventeen adherence scales were identified in primary care, most of which targeted a single pathology, especially hypertension. The most cited scale is the MMAS Morisky medication adherence scale. Three scales were developed for patients with multiple chronic diseases. One scale was developed for patients older than 65 years—the Strathclyde compliance risk assessment tool (SCRAT)—and two scales were developed for adult patients whatever their age—the instrument developed by Sidorkiewicz et al., and the DAMS, diagnostic adherence to medication scale.

**Conclusion:** Two scales have been developed and validated in primary care to assess patient adherence with multiple chronic diseases: the DAMS and the instrument developed by Sidorkiewicz et al. A simple, reliable, reproducible primary care scale would assess the impact of actions developed to improve adherence: motivational interviewing, patient therapeutic education, and the ASALÉE protocol.

# Abstract: Forms and frequency of sanctions in primary healthcare in Macedonia

**DOI:** 10.1080/13814788.2019.1643166

**Authors:** Marija Zafirovska, Ljubin Šukriev, Katerina Kovačević, Marta Tundževa

**Affiliations:** Ss Cyril and Methodius University - Faculty of Medicine - Center of Family Medicine, Ss Cyril and Methodius University, Skopje, Macedonia

## Abstract

**Background:** Regulating the quality and effectiveness of the work of general practitioners is essential for a sound healthcare system. In the Republic of Macedonia this is regulated by the Health Insurance Fund through a system of penalties/sanctions.

**Research question:** The goal of this study is to evaluate the types and effectiveness of the sanctions used on primary care practitioners.

**Methods:** This is a quantitative research study for which we used an anonymous survey with 18 questions. This survey was distributed to 443 randomly selected general practitioners from different parts of Macedonia and 438 of them responded. For the quantitative data, we used the Pearson’s chi-squared test, correlation and descriptive statistics. Part of the survey is qualitative, consisting of comments and opinions of the general practitioners.

**Results:** From the participants, 336 were female and 102 were male. The doctors’ gender was not associated with sanctioning. Most general practitioners were in the age categories of 30–39 and 40–49 years. The participants’ age had a significant influence on sanctioning—older doctors were sanctioned more frequently. Out of 438 participants, 33.3% were specialists in family medicine and 66.7% general practitioners. Specialists in family medicine were sanctioned significantly more frequently than general practitioners. Doctors that worked in the hospital or 19 km from the nearest hospital were significantly more frequently sanctioned. The three most common reasons for sanctions were financial consumption of prescriptions and referrals above the agreed amount, higher rate of sick leaves and/or justification of sick leaves and unrealized preventative goals or education. ‘Financial sanction by scale’ was the most common type of sanction: 49.8% of participants. Doctors who followed the guidelines, but who were exposed to violence were sanctioned significantly more frequently.

**Conclusion:** We can observe that age, speciality, the distance of the workplace from the nearest hospital and violence influence sanctioning.

# Abstract: The Hawthorne effect: Systematic review and meta-analysis

**DOI:** 10.1080/13814788.2019.1643166

**Authors:** Christophe Berkhout, Ornella Berbra, Jonathan Favre, Lieve Peremans, Paul Van Royen

**Affiliations:** Medical School, Department of General Medicine, Lille, France

## Abstract

**Background:** Biases are major barriers to external validity of studies, reducing evidence. Among these biases, the definition and the reality of the Hawthorne effect (HE) (or observation bias) remains controversial. According to McCambridge in a review from 2013, the Hawthorne effect is a behaviour change occurring when the subject is being observed during a scientific study. This effect would be multifactorial, and he suggests the term ‘effects of research participation.’ However, the reviewed studies were conflicting and evidence is sparse.

**Research question:** We updated McCambridge’s review to actualize the definition of the HE.

**Methods:** McCambridge’s most recent article dated back to January 3, 2012. We focused on the articles published between January 1, 2012 and August 10, 2018 searching Medline. We used the sole keyword ‘Hawthorne Effect.’ The search was filtered based on the dates, the availability of an abstract and the languages English and French. We included articles defining or evaluating the HE. Articles citing the effect without defining it or irrelevant to the topic were excluded. Two independent readers searched and analysed the articles. Discrepancies were solved by consensus.

**Results:** Out of 106 articles, 42 articles were included. All the articles acknowledged an observation bias, considered as significant or not, depending on the population (education, literacy), the methods and the variable of interest. It was a psychological change, limited in time. The HE was defined as a change of behaviour related to direct or indirect observation of the subjects or the investigators, to their previous selection and commitment in the study (written agreement) and to social desirability. Despite observations, articles were conflicting. Some do confirm the existence of the HE, others deny it. Meta-analysis is ongoing.

**Conclusion:** No formal consensus regarding the definition of the effect has been reached so far. However, the authors agree on its implication as an experimental artefact.
